# Antenatal corticosteroid therapy (ACT) and size at birth: A population-based analysis using the Finnish Medical Birth Register

**DOI:** 10.1371/journal.pmed.1002746

**Published:** 2019-02-26

**Authors:** Alina Rodriguez, Yingbo Wang, Anohki Ali Khan, Rufus Cartwright, Mika Gissler, Marjo-Riitta Järvelin

**Affiliations:** 1 Department of Epidemiology and Biostatistics, Imperial College London, London, United Kingdom; 2 School of Psychology, University of Lincoln, Lincoln, United Kingdom; 3 Department of Obstetrics and Gynaecology, Oxford University Hospitals NHS Trust, Oxford, United Kingdom; 4 THL National Institute for Health and Welfare, Information Services Department, Helsinki, Finland; 5 Karolinska Institute, Department of Neurobiology, Care Sciences and Society, Division of Family Medicine, Stockholm, Sweden; 6 Unit of Primary Health Care, Oulu University Hospital, Oulu, Finland; 7 Biocenter Oulu, University of Oulu, Oulu, Finland; 8 Center for Life Course Health Research, Faculty of Medicine, University of Oulu, Oulu, Finland; 9 Department of Life Sciences, College of Health and Life Sciences, Brunel University London, London, United Kingdom; London School of Hygiene and Tropical Medicine, UNITED KINGDOM

## Abstract

**Background:**

Antenatal corticosteroid therapy (ACT) is used clinically to prepare the fetal lung for impending preterm birth, but animal and human studies link corticosteroids to smaller birth size. Whether ACT is associated with birth size is debated; therefore, we assessed differences in birth size in treated versus untreated pregnancies.

**Methods and findings:**

This observational register-based study used data from the Finnish Medical Birth Register (FMBR) covering all births in Finland (January 1, 2006–December 31, 2010). We used unadjusted and adjusted regression analyses as well as propensity score matching (PSM) to analyze whether birth size differed by ACT exposure. PSM provides a stringent comparison, as subsamples were created matched on baseline and medical characteristics between treated and untreated women. All analyses were stratified by timing of birth. The primary study outcome was birth size: birth weight (BWT), birth length (BL), ponderal index (PI), and head circumference (HC) measured immediately after birth and recorded in the FMBR. Additional analyses explored indicators of neonatal health in relation to ACT exposure and birth size. A total of 278,508 live-born singleton births with ≥24 gestational completed weeks were registered in the FMBR during the 5-year study period. Over 4% of infants were born preterm, and 4,887 women were treated with ACT (1.75%). More than 44% of the exposed infants (*n* = 2,173) were born at term. First, results of unadjusted regression analyses using the entire sample showed the greatest reductions in BWT as compared to the other analytic methods: very preterm −61.26 g (±SE 24.12, *P* < 0.01), preterm −232.90 g (±SE 17.24, *P* < .001), near term −171.50 g (±SE 17.52, *P* < .001), and at term −101.95 g (±SE 10.89, *P* < .001). Second, using the entire sample, regression analyses adjusted for baseline and medical conditions showed significant differences in BWT between exposed and unexposed infants: very preterm −61.54 g (±SE 28.62, *P* < .03), preterm −222.78 g (±SE 19.64, *P* < .001), near term −159.25 g (±SE 19.14, *P* < .001), and at term −91.62 g (±SE 11.86, *P* < .03). Third, using the stringent PSM analyses based on matched subsamples, infants exposed to ACT weighed less at birth: −220.18 g (±SE 21.43, *P* < .001), −140.68 g (±SE 23.09, *P* < .001), and −89.38 g (±SE 14.16, *P* < .001), born preterm, near term, and at term, respectively. Similarly, significant reductions in BL and HC were also observed using the three analytic methods. There were no differences among postterm infants regardless of analytic method. Likewise, we observed no differences with respect to PI. Additional analyses showed that exposed and unexposed infants had generally similar Apgar scores at birth, yet the ACT-treated infants received greater medical care during the first 7 days of life and beyond. Our study is mainly limited by lack of data in FMBR specifying the interval between treatment and birth as well as other potential confounders that could not be tested.

**Conclusions:**

In this study, ACT was consistently associated with reduction in birth size for infants born preterm, near term, or at term. Further investigation is warranted alongside reevaluation of guidelines. Efforts need to be made to correctly identify and target patients who will deliver preterm. Reduced growth should be considered when deliberating early care decisions.

## Abstract 2

Antenatal Corticosteroid Therapy (ACT) is used clinically to prepare the fetal lung for impending preterm birth but may cause smaller birth size. In this national registry study, Alina Rodriguez and colleagues measure this association according to timing of birth.

## Author summary

**Why was this study done?:**

- Presently, guidelines recommend one dose, repeated over 24 hours, of antenatal corticosteroid therapy (ACT) to accelerate fetal lung maturation in cases of threatened preterm birth in an effort to reduce infant mortality related to respiratory distress.
- Many exposed infants are born at term and thus exposed unnecessarily to potential harms of ACT.
- There is persistent concern about the potential risk of ACT to decrease birth size, but previous results in humans are uncertain.
- Most studies do not use birth size as the primary outcome and do not examine all measurements, i.e., birth weight (BWT), birth length (BL), head circumference (HC), and ponderal index (PI).

**What did the researchers do and find?:**

- The entire population of singleton and live-born infants in Finland was studied during a 5-year period (January 1, 2006–December 31, 2010)—in total, 278,508 infants.
- Propensity score was used to generate matched subgroups of ACT-exposed and ACT-unexposed infants to compare size at birth.
- Exposure to ACT is consistently associated with smaller size at birth including BWT, BL, and HC among infants born preterm, near term, and at term, but not with PI in propensity score–matched analyses.
- Deficits in birth size in relation to ACT were greatest for infants born preterm regardless of analytic strategy.

**What do the findings mean?:**

- There was a clinically significant reduction in birth size among infants exposed to ACT and born at term—i.e., unnecessarily exposed—as compared to unexposed counterparts.
- ACT-exposed infants received more medical care than matched unexposed counterparts, counter to expectation, suggesting that treated infants’ health was more compromised at birth and required more medical intervention and that the expected prophylactic effect of ACT was not fully realized.
- Early care decisions need to identify high-risk patients and weigh benefits of ACT against potential harm of unnecessary exposure.
- These findings provide strong evidence indicating ACT is associated with reduced fetal growth in humans and provide an agenda for further studies and on par with reexamination of guidelines.

## Introduction

Complications arising from preterm birth are the primary cause of mortality in children under 5 years old [1] and a leading cause of morbidity across the life course [2]. Antenatal corticosteroid therapy (ACT) accelerates maturation of fetal lung tissue. ACT is used prophylactically when preterm birth is threatened to reduce risk of respiratory distress syndrome (RDS), associated morbidity, and mortality in preterm infants [3]. International guidelines [4–6] recommend that women between 24 and 34 weeks of gestation who are at risk of preterm delivery within 7 days should receive ACT. However, recent reports show that a large portion of women receive ACT inappropriately [7], with optimal timing of treatment administered in only 40.8% of patients [8]. Moreover, the estimated number needed to treat with ACT to prevent one case of RDS varies widely [9–10]. As ACT is often initiated before a diagnosis of preterm birth is confirmed, and given the uncertainty in predicting parturition, as many as 40% [7–10] of infants exposed to ACT go on to be delivered at term. These infants would not be expected to develop RDS, with or without ACT, but have been exposed to any potential harms [11–13], thus raising concerns about risks.

ACT may disturb fetal growth [14] and consequently increases risk of disease across the life course [15–17]. Concern stems from experimental animal models that demonstrate that glucocorticoid administration (at clinically bioequivalent doses) impairs fetal growth and development of skeletal muscle as well as other organ tissues, which leads to a wide range of dysfunctions in the cardiovascular, metabolic, endocrine, nervous, and reproductive systems in the adult offspring [17]. According to the systematic review focusing on birth size [14], the evidence in humans for disturbed fetal growth is only suggestive. Firm conclusions could not be drawn because of the studies’ design limitations, particularly inadequate control for confounders [14]. Because of the widespread use of ACT [18], it is especially critical that there is clear understanding of potential adverse effects related to fetal growth when making early care decisions. Most available studies have not controlled for relevant factors, are not large enough to provide robust estimates, or do not use birth size as the primary outcome. Existing evidence [19] is graded as moderate quality by the World Health Organization.

Our objective was to address previous shortcomings and investigate the association between ACT exposure and birth size—birth weight (BWT), birth length (BL), ponderal index (PI), and head circumference (HC)—using data from the nationwide Finnish Medical Birth Register (FMBR). Because we had complete country data available, we were able to specifically examine carefully matched pregnancies to address issues of confounding and examine whether ACT was related to birth size of infants born at term, i.e., who received the prophylactic treatment unnecessarily. Given the very large sample size, we set out to create balanced subgroups (treated versus untreated) for direct comparison, using propensity score matching (PSM). The subgroup matching took into account factors influencing ACT treatment. Additional analyses were conducted to explore whether indices of poor fetal health, potentially precipitating ACT treatment, were related to growth deficits. We hypothesized that ACT-exposed infants would be smaller at birth than unexposed infants in one or more of the birth size values.

## Methods

### Study oversight

The National Institute of Health and Welfare (https://thl.fi/en/web/thlfi-en/about-us) in Finland, in accordance with national data protection legislation, authorized the release of the anonymized data for this register-based study and provided ethical approval. Individual informed consent was not required, as only deidentified data were used without any additional patient contact. This study is reported as per the Strengthening the Reporting of Observational Studies in Epidemiology (STROBE) guideline (S1 Checklist).

### Study population

We use prospective data registered in FMBR, covering all births within the period January 1, 2006, to December 31, 2010, in Finland. Antenatal care in Finland is tax-paid and offered without fees to all women residing in the country. Standardized information is recorded prospectively during antenatal, obstetric, and neonatal care for all pregnant women and neonates in all clinics and hospitals in Finland. These data are copied after delivery and sent to FMBR for archiving. The FMBR includes data on all mothers and their live-born infants and stillbirths weighing ≥500 g or with a gestational age of ≥22 weeks.

**Fig 1:**
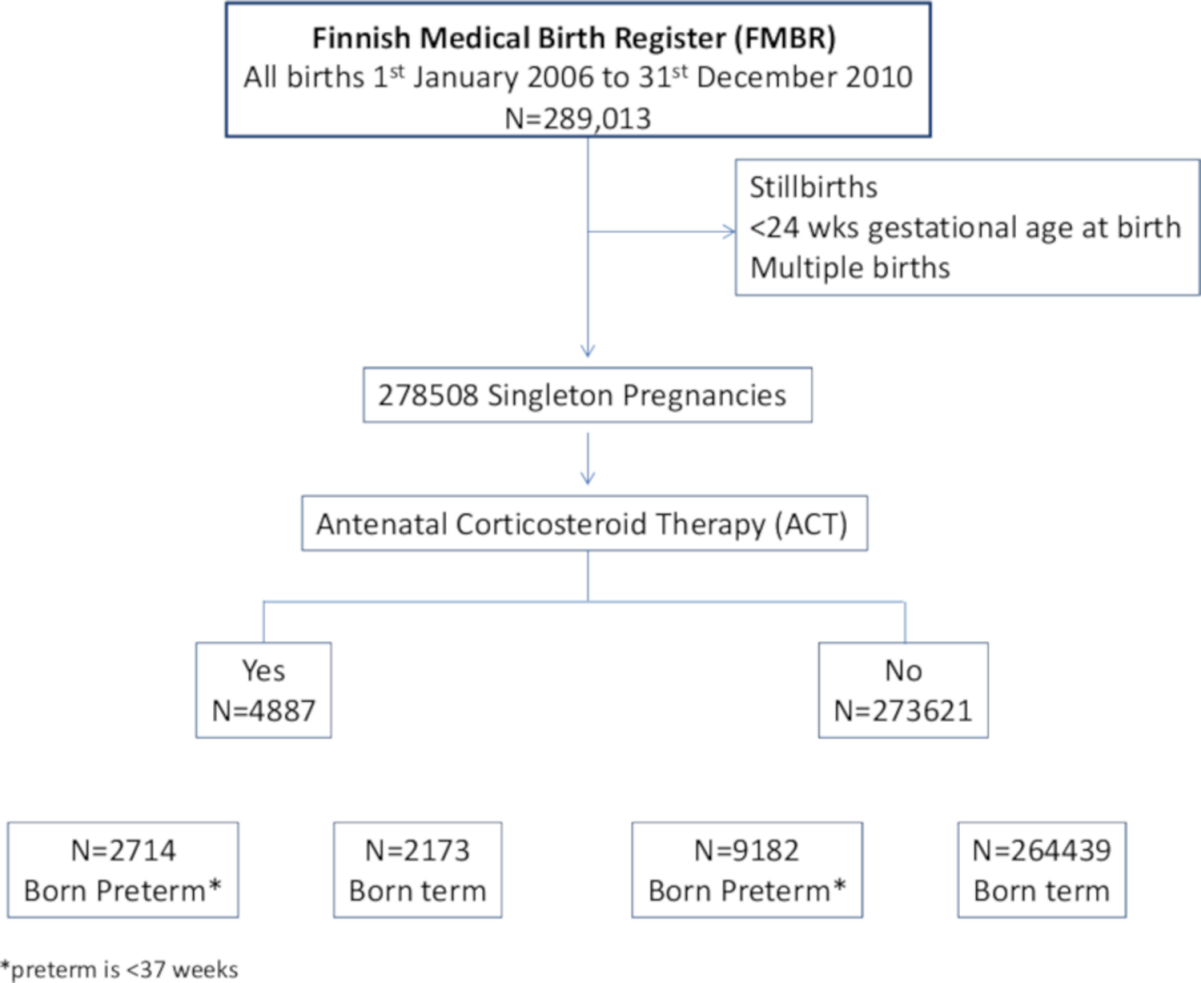
Pregnancies, ACT treatment, and timing of births. ACT, antenatal corticosteroid therapy.

The total number of births recorded during the study period was 289,722. We excluded births <24 gestational weeks, multiple births (because of risk of decreased birth size), and stillbirths. Fig 1 shows the entire study population from which we drew the subsample of matched participants for the main analysis. Results for the entire sample are presented in the supporting information.

### ACT

ACT administration during the current pregnancy was recorded in FMBR as either received treatment or not. The recommended treatment consisted of betamethasone 12 mg administered twice, 24 hours apart. According to national guidelines, delivery can be postponed through tocolytic therapy to enable corticosteroid prophylaxis and referral to tertiary hospital, if necessary.

### Birth size

Using a uniform method across Finland, medical staff measure BWT accurate to ±1 g, BL, and HC in centimeters immediately after birth. We calculated PI using the standard formula (BWT [kg] / BL [m^3^]).

### Confounding factors

The most important confounder is timing of birth, as the length of gestation directly sets the limits or opportunity for fetal growth. We therefore examined the impact of ACT on birth size stratified by the timing of birth: very preterm (gestational weeks 24–29), preterm (30–34), near term (35–37), at term (38–41), and postterm (≥42). Gestational age at birth is recorded in FMBR as the best estimate including last menstrual period and ultrasound examination. Women routinely undergo an early-pregnancy ultrasound scan by a specially trained midwife at maternity outpatient clinics across the country between 11 + 0 and 13 + 6 weeks of pregnancy. This scan is used to date pregnancies and is offered free of charge to all women registered at the antenatal clinics. Uptake is voluntary, and the majority use the service [20].

**Fig 2:**
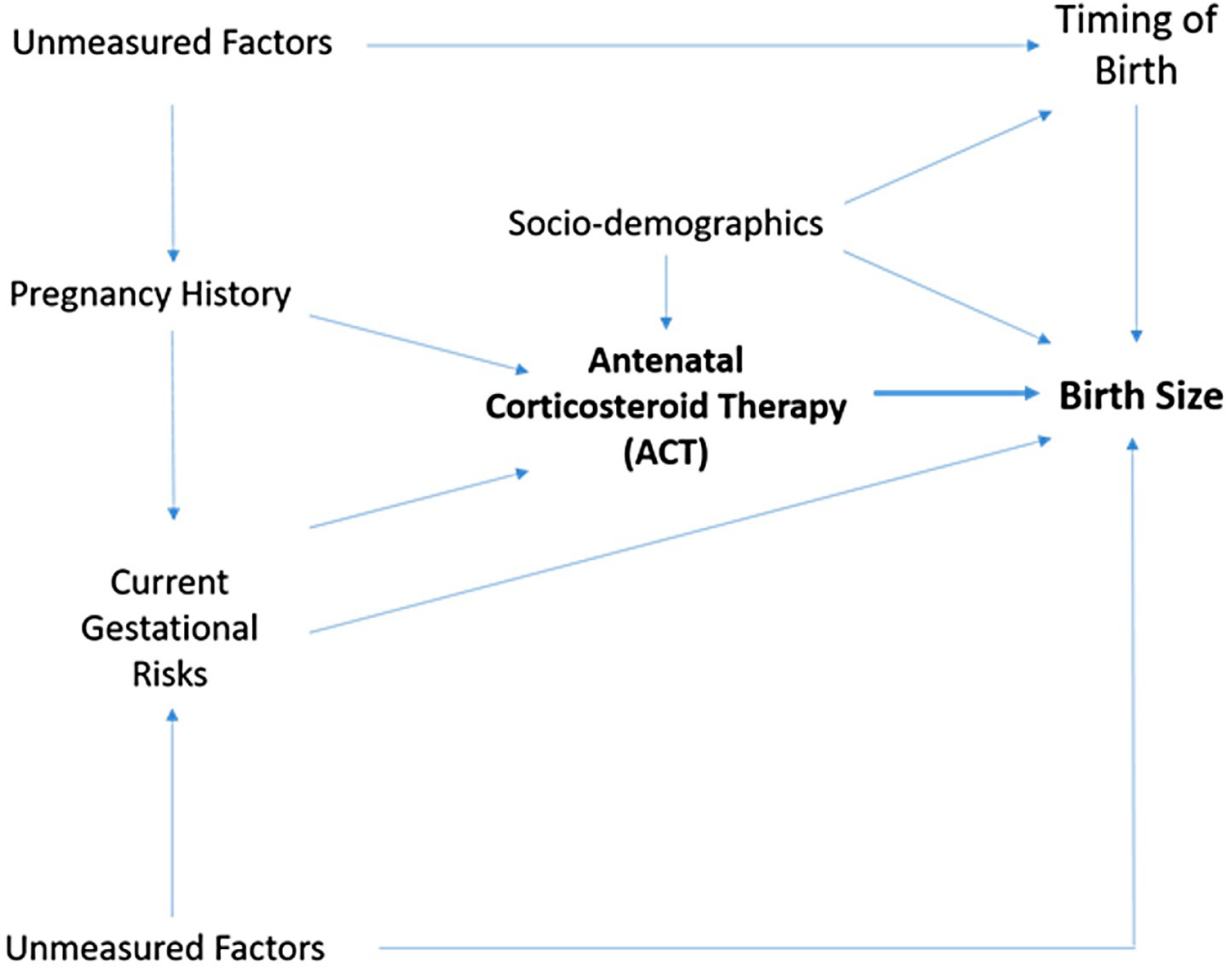
DAG illustrating factors potentially influencing birth size. DAG, directed acyclic graph.

The FMBR includes information of clinical relevance on sociodemographics, pregnancy medical history, and gestational complications, which we used as potential confounders, depicted in Fig 2 and listed in Table 1. These variables were adjusted for in multiple regression analyses using the full sample. In the main analysis, the confounders were used to create a propensity score–matched subset of participants for matched comparison of exposed and unexposed infants.

**Table 1:** Characteristics of study population available in the FMBR and inclusion in PSM.

Variables		Treated (*n* = 4,887)	Control (*n* = 273,621)	Included in PSM
Maternal demographics				
Age (years)		29.9 ± 5.79	29.6 ± 5.33	Yes
Social economic status^a^		47.6 ± 16.60	46.7 ± 16.26	Yes
Cohabitation	Yes	4,604 (94.2%)	258,624 (94.5%)	Yes
	No	283 (5.8%)	14,997 (5.5%)	
Pregnancy characteristics				
Height (cm)		165.0 ± 6.32	165.6 ± 6.01	Yes
Prepregnancy weight (kg)		65.8 ± 14.70	66.6 ± 13.83	Yes
Prepregnancy BMI		24.2 ± 5.15	24.2 ± 4.73	Yes
Number of previous pregnancies		1.7 ± 2.10	1.5 ± 1.80	Yes
Number of miscarriages		0.4 ± 0.89	0.3 ± 0.66	Yes
Number of induced abortions		0.2 ± 0.55	0.2 ± 0.47	Yes
Number of ectopic pregnancies		0.0 ± 0.19	0.0 ± 0.15	Yes
Medical risk factors				
Ovum donation	Yes	252 (5.2%)	5,199 (1.9%)	Yes
	No	4,635 (94.8%)	268,422 (98.1%)	
Insemination	Yes	87 (1.8%)	1,977 (0.7%)	Yes
	No	4,800 (98.2%)	271,644 (99.3%)	
Glucose test performed	Yes	1,746 (35.7%)	102,356 (37.4%)	Yes
	No	3,141 (64.3%)	171,265 (62.6%)	
Glucose test pathological	Yes	510 (10.4%)	26,317 (9.6%)	Yes
	No	4,377 (89.6%)	247,304 (90.4%)	
Insulin treatment	Yes	156 (3.2%)	5,260 (1.9%)	Yes
	No	4,731 (96.8%)	268,361 (98.1%)	
Self-reported smoking	Yes	899 (18.9%)	39,505 (14.8%)	Yes
	No	3,756(81.1%)	227,420(85.2%)	
Hospitalization due to high BP	Yes	319 (6.5%)	5,798 (2.1%)	Yes
	No	4,568 (93.5%)	267,823 (97.9%)	
Infant characteristics				
Sex	Male	2,584 (52.9%)	140,032 (51.2%)	Yes
	Female	2,303 (47.1%)	133,589 (48.8%)	
Gestational age at birth (weeks)		35.0 ± 4.31	39.5 ± 1.50	
Mode of delivery				
Vaginal		2,977 (60.9%)	231,504 (84.6%)	
Assisted vaginal delivery		610 (12.5%)	17,114 (6.3%)	
Cesarean section		1,300 (26.6%)	25,003 (9.1%)	
Birth weight (g)		2,570.9 ± 1,001.9	3,546.2 ± 503.56	
Birth length (cm)		45.6 ± 5.52	50.2 ± 2.17	
Ponderal index		2.6 ± 0.45	2.8 ± 0.36	
Head circumference (cm)		32.4 ± 3.45	35.0 ± 1.48	
Apgar score				
1 minute		7.8 ± 2.05	8.6 ± 1.19	
5 minutes		8.2 ± 1.67	9.1 ± 0.89	
Days in hospital		6.1 ± 11.58	3.2 ± 2.38	

^a^According to the national classification of socioeconomic status: https://www.stat.fi/meta/luokitukset/sosioekon_asema/001-1989/index_en.html.

Abbreviations: BP, blood pressure; FMBR, Finnish Medical Birth Register; PSM, propensity score matching.

### Factors used in additional analyses

We examined the factors available in FMBR that could be used as indicators of neonatal health to shed light on whether any growth restriction was due to ACT or to poor fetal health, which would both limit growth and precipitate treatment.

The Apgar score was implemented in 1952 as a quick way to evaluate the physical condition of the newborn by assessing heart rate, respiratory effort, muscle tone, reflex irritability, and color. The Apgar score conducted at 5 minutes after birth has been found to be an excellent predictor of infant survival in the first 28 days of life and thus considered a meaningful measure of neonatal health [21].

Neonatal care is recorded in FMBR for the first 7 days of life. We used these data to compare ACT-exposed and ACT-unexposed infants. Data were also available indicating whether infants were still hospitalized beyond 7 days.

Lastly, we present in the supporting information results by mode of delivery. Medically indicated preterm labor reflects a complication with the mother or fetus and occurs mostly because of preeclampsia, placental abruption or dysfunction, fetal distress, and fetal growth restriction [22]. In contrast, spontaneous preterm labor starts because of physiologic changes in the cervix, uterus, or amniotic sac. Causal risk factors associated with spontaneous preterm birth include intrauterine infections, psychosocial stress, sociodemographic characteristics, and maternal illness [23]. About 70% of preterm births are considered spontaneous [22–23]. Planned cesarean at term is typical for breech presentation, whereas unplanned or emergency is common for fetal distress.

### Statistical analyses

All statistical analyses were planned a priori and carried out using SAS software, version 9.4 (SAS Institute). Our a priori thinking is depicted in Fig 2, which illustrates the potential causal pathways in a directed acyclic graph (DAG) that guided our work. All models were stratified by timing of birth. ACT as a single exposure was examined for each of the four birth size outcomes stratified by five categories of gestational age at birth. Randomized controlled trials (RCTs) provide firm evidence of causal associations. However, the risk of bias is not completely eliminated. Analytic strategies in RCTs do not always realistically reflect clinical complexity or report potential biases. We set out to examine a very large number of pregnancies in order to be able to carry out matched comparisons. The purpose of random assignment is to equate participants in the treatment and control groups in order to isolate the effectiveness of treatment. However, outside of an RCT, this equality among participants is not known, because specific characteristics can predispose individuals to either receive treatment or not. In such a case, the treatment and control groups are different; thus, the treatment effect cannot be isolated, and firm conclusions cannot be drawn about the causal effect of treatment. PSM technique attempts to equate the treatment and control groups after allocation in an observational study in an effort to produce equal groups. We adopted the propensity score approach to match participants and minimize the imbalance with regards to the confounding factors. We match subsamples on all data archived in the FMBR and thus available for analysis in this study. The two groups (treated versus control) were carefully balanced on baseline characteristics associated with treatment allocation, thereby reducing confounding associated with receipt of treatment. Our a priori strategy was to conduct stratified unadjusted and adjusted regression analyses using the entire sample (presented in the supporting material), followed by the formation of matched subsamples and analysis by stratified regression models using PSM score (presented as the main results). In this way, we can compare results using two analytic strategies and are able to ascertain whether any observed differences are driven by bias.

Because all of the background and medical variables recorded in FMBR bear clinical relevance, all were included as confounders, including, e.g., those as identified in previous work such as cohabitation with expectant father [24–25]. Thus, all available background and medical factors recorded in FMBR were used. Few missing values are present in the dataset, and in such cases, pairwise deletions were made. We analyzed the entire cohort using multiple regressions to calculate the association between ACT exposure and birth size (BWT, BL, PI, and HC) and in the fully adjusted model including all the confounders. We then used the confounders to generate propensity scores to match ACT patients with controls. ACT patients were matched to controls on the logit propensity score, using “nearest neighbor matching” [26]. The large sample size permitted a subsample to be generated for analysis consisting of a matching ratio of 5:1, controls to patients, which has been shown to be the optimum matching ratio [27]. However, because relatively few infants are born very preterm, we used 1:1 matching for this stratum. The main analysis comprised direct comparison in the matched samples consisting of 3,077 infants prenatally exposed to ACT (treated) and 11,821 unexposed infants (control).

Lastly, we performed additional analyses to assess whether potential differences in birth size could be attributed to poorer health, as indexed by available data, among the exposed versus unexposed infants. We examined Apgar scores at 5 minutes (additionally stratified by infant sex, as sex differences have been previously reported), neonatal care, and mode of delivery.

## Results

There were 278,508 live-born singleton births recorded in Finland during the study period. Of these, 11,896 (4.27%) infants were born preterm, 4,887 women were treated with ACT (1.75%), and 44% of the exposed infants (*n* = 2,173) were born at term, as shown in Fig 1.

Baseline characteristics of the study population and current medical factors archived in FMBR are listed in Table 1. These factors were used as the confounders for adjustment in the regression analyses of the entire sample and used for the propensity score calculation.

Results of unadjusted and adjusted regression analyses using the entire population are presented in S1 Table. The results showed very consistent deficits in birth size, though estimates were slightly attenuated in the adjusted models. ACT-exposed infants born very preterm were lighter by 61 g and shorter by approximately .69 cm. Infants born preterm who had been exposed to ACT were more than 200 g lighter than unexposed counterparts. These infants also were significantly shorter and thinner (PI) and had a smaller HC. Near-term and term infants exposed to ACT were also significantly lighter and smaller, but not thinner, in both unadjusted and adjusted comparisons. Postterm infants showed no differences in size regardless of ACT exposure.

Table 2 presents results evaluating birth size differences between ACT exposure and controls by way of PSM subsample analysis. As in the full sample analysis, infants who had been treated with ACT and born preterm showed the biggest deficits in birth size as compared to other gestational categories. Preterm infants were 220 g lighter and 1.43 cm shorter, had a .92 cm smaller HC, and were significantly thinner (PI). ACT-exposed infants born near term and at term, respectively, were significantly lighter (140 g, 89 g) and shorter (.71 cm, .36 cm) and had smaller HC (.39 cm, .21 cm) but were not thinner. Postterm infants were of similar size whether ACT treated or not.

**Table 2:** Comparison of birth size by ACT treatment versus no treatment using PSM subsamples stratified by timing of birth.

Timing of Birth	Measurements	Number Treated	Number Control	Point Estimate	SE	*P*
Very preterm	BWT (g)	144	144	−43.45	34.90	0.22
	BL (cm)	121	121	−0.22	0.44	0.61
	PI	120	120	−0.06	0.06	0.32
	HC (cm)	56	56	0.35	0.44	0.43
Preterm	BWT (g)	901	1,521	−220.18	21.43	< .001
	BL (cm)	789	1,285	−1.43	0.15	< .001
	PI	789	1,285	−0.05	0.02	0.01
	HC (cm)	643	995	-0.92	0.10	< .001
Near term	BWT (g)	685	3,425	−140.68	23.09	< .001
	BL (cm)	667	3,282	−0.71	0.11	< .001
	PI	667	3,282	−0.02	0.01	0.24
	HC (cm)	647	3,141	−0.39	0.07	< .001
Term	BWT (g)	1,301	6,504	−89.38	14.16	< .001
	BL (cm)	1,291	6,431	−0.36	0.06	< .001
	PI	1,291	6,431	−0.01	0.01	0.24
	HC (cm)	1,279	6,326	−0.21	0.04	< .001
Postterm	BWT (g)	45	225	26.46	76.01	0.73
	BL (cm)	45	225	−0.28	0.29	0.34
	PI	45	225	0.06	0.03	0.10
	HC (cm)	45	223	0.04	0.22	0.86

very preterm = gestational weeks 24–29; preterm = gestational weeks 30–34; near term = gestational weeks 35–37; term = gestational weeks 38–41; postterm = gestational weeks 42+

Abbreviations: ACT, antenatal corticosteroid therapy; BL, birth length; BWT, birth weight; HC, head circumference; PI, ponderal index; PSM, propensity score matching.

Taken together, significant differences were detected by all analytic strategies and for the birth size measurements we tested, i.e., BWT, BL, and HC. ACT-exposed infants were not thinner with respect to length—i.e., no consistent differences in PI. The results using the entire sample were in the same direction as the PSM subsample results, though the reductions in growth were greater using the complete sample. As compared to controls, ACT-exposed infants born between gestational weeks 30 and 41 were consistently smaller in birth size regardless of analytic strategy.

Additional analyses were conducted to assess whether indicators of health at birth differed by ACT exposure. Table 3 shows that Apgar scores at 5 minutes were somewhat inconsistent across groups. ACT-exposed and unexposed boys showed no differences in Apgar scores, with the exception of higher Apgar among unexposed, term-born males. In contrast, there were some differences in Apgar scores for girls. ACT was associated with lower scores for those born near term but higher scores for those born at or post term as compared to their unexposed counterparts.

**Table 3:** Comparison of 5-minute Apgar score by ACT for the PSM subsamples stratified by timing of birth and infant sex.

Sex	Birth	Treated Mean (SD) *N*	Control Mean (SD) *n*	Two-sample *t* test *P*
Male	Very preterm	6.0 (2.13) 75	5.8 (2.72) 76	0.5889
	Preterm	8.1 (1.58) 415	7.8 (2.09) 791	0.2338
	Near term	8.5 (1.30) 278	8.8 (1.18) 1,536	0.3847
	Term	8.9 (1.11) 510	9.1 (0.76) 2,937	0.0231
	Postterm	8.8 (1.21) 13	9.1 (0.80) 92	0.2811
Female	Very preterm	6.9 (1.76) 63	6.1 (2.15) 56	0.0577
	Preterm	7.9 (1.64) 328	7.9 (1.97) 564	0.3652
	Near term	8.6 (1.23) 258	8.9 (1.10) 1,486	0.0264
	Term	9.2 (0.79) 453	9.1 (0.84) 2,763	0.0058
	Postterm	9.5 (0.52) 13	9.0 (0.67) 102	0.003

preterm = gestational weeks 24–29; preterm = gestational weeks 30–34; near term = gestational weeks 35–37; term = gestational weeks 38–41; postterm = gestational weeks 42+

Abbreviations: ACT, antenatal corticosteroid therapy; PSM, propensity score matching.

ACT-exposed infants received significantly more care after birth than controls (Table 4). As seen in Table 4, there were generally no differences by ACT in medical care provided to the very preterm and postterm groups, though untreated very preterm infants were more likely to receive hospital transfer. Preterm, near-term, and term-born infants exposed to ACT had higher rates of respirator care and intubation than matched infants. Further, Table 5 shows that infants hospitalized beyond the seventh day clearly were more likely to be from the ACT group born near term or at term.

**Table 4:** Inpatient treatment during first 7 days of life by ACT for PSM subsamples stratified by timing of birth.

Birth	Treatment	Received	Treated	Control	*P*
Very preterm	NICU	Yes	141 (97.2%)	131 (90.3%)	
		No	4 (2.8%)	14 (9.7%)	0.026
	Hospital transfer	Yes	2 (1.4%)	13 (9.0%)	
		No	143 (98.6%)	132 (91.0%)	0.006
	Respirator care	Yes	106 (73.1%)	107 (73.8%)	
		No	39 (26.9%)	38 (26.2%)	1
	Intubation	Yes	70 (48.3%)	68 (46.9%)	
		No	75 (51.7%)	77 (53.1%)	0.906
	Blood transfusion	Yes	14 (9.7%)	12 (8.3%)	
		No	131 (90.3%)	133 (91.7%)	0.838
	Light therapy	Yes	66 (45.5%)	76 (52.4%)	
		No	79 (54.5%)	69 (47.6%)	0.29
	Antibiotic treatment	Yes	133 (91.7%)	116 (80.0%)	
		No	12 (8.3%)	29 (20.0%)	0.006
	Vitamin K	Yes	132 (91.0%)	118 (81.4%)	
		No	13 (9.0%)	27 (18.6%)	0.026
	BCG vaccination	Yes	2 (1.4%)	4 (2.8%)	
		No	143 (98.6%)	141 (97.2%)	0.684
	Hypothyroidism screening	Yes	134 (92.4%)	126 (86.9%)	
		No	11 (7.6%)	19 (13.1%)	0.176
	Metabolic disorder screening	Yes	0	0	
		No	145 (100.0%)	145 (100.0%)	1
Preterm	NICU	Yes	804 (89.2%)	1,362 (89.5%)	
		No	97 (10.8%)	159 (10.5%)	0.838
	Hospital transfer	Yes	87 (9.7%)	84 (5.5%)	
		No	814 (90.3%)	1,437 (94.5%)	< .001
	Respirator care	Yes	188 (20.9%)	231 (15.2%)	
		No	713 (79.1%)	1,290 (84.8%)	< .001
	Intubation	Yes	89 (9.9%)	112 (7.4%)	
		No	812 (90.1%)	1,409 (92.6%)	0.033
	Blood transfusion	Yes	28 (3.1%)	16 (1.1%)	
		No	873 (96.9%)	1,505 (98.9%)	< .001
	Light therapy	Yes	427 (47.4%)	818 (53.8%)	
		No	474 (52.6%)	703 (46.2%)	0.002
	Antibiotic treatment	Yes	638 (70.8%)	800 (52.6%)	
		No	263 (29.2%)	721 (47.4%)	< .001
	Vitamin K	Yes	851 (94.5%)	1,345 (88.4%)	
		No	50 (5.5%)	176 (11.6%)	< .001
	BCG vaccination	Yes	32 (3.6%)	81 (5.3%)	
		No	869 (96.4%)	1,440 (94.7%)	0.047
	Hypothyroidism screening	Yes	855 (94.9%)	1,427 (93.8%)	
		No	46 (5.1%)	94 (6.2%)	0.282
	Metabolic disorder screening	Yes	1 (0.1%)	8 (0.5%)	
		No	900 (99.9%)	1,513 (99.5%)	0.167
Near term	NICU	Yes	288 (42.0%)	959 (28.0%)	
		No	397 (58.0%)	2,466 (72.0%)	< .001
	Hospital transfer	Yes	27 (3.9%)	65 (1.9%)	
		No	658 (96.1%)	3,360 (98.1%)	0.003
	Respirator care	Yes	25 (3.6%)	75 (2.2%)	
		No	660 (96.4%)	3,350 (97.8%)	0.029
	Intubation	Yes	23 (3.4%)	57 (1.7%)	
		No	662 (96.6%)	3,368 (98.3%)	0.006
	Blood transfusion	Yes	6 (0.9%)	7 (0.2%)	
		No	679 (99.1%)	3,418 (99.8%)	0.013
	Light therapy	Yes	224 (32.7%)	835 (24.4%)	
		No	461 (67.3%)	2,590 (75.6%)	< .001
	Antibiotic treatment	Yes	134 (19.6%)	361 (10.5%)	
		No	551 (80.4%)	3,064 (89.5%)	< .001
	Vitamin K	Yes	671 (98.0%)	3,362 (98.2%)	
		No	14 (2.0%)	63 (1.8%)	0.757
	BCG vaccination	Yes	77 (11.2%)	513 (15.0%)	
		No	608 (88.8%)	2,912 (85.0%)	0.01
	Hypothyroidism screening	Yes	664 (96.9%)	3,284 (95.9%)	
		No	21 (3.1%)	141 (4.1%)	0.236
	Metabolic disorder screening	Yes	20 (2.9%)	66 (1.9%)	
		No	665 (97.1%)	3,359 (98.1%)	0.107
Term	NICU	Yes	128 (9.8%)	438 (6.7%)	
		No	1,173 (90.2%)	6,067 (93.3%)	< .001
	Hospital transfer	Yes	16 (1.2%)	39 (0.6%)	
		No	1,285 (98.8%)	6,466 (99.4%)	0.018
	Respirator care	Yes	11 (0.8%)	20 (0.3%)	
		No	1,290 (99.2%)	6,485 (99.7%)	0.012
	Intubation	Yes	14 (1.1%)	28 (0.4%)	
		No	1,287 (98.9%)	6,477 (99.6%)	0.011
	Blood transfusion	Yes	1 (0.1%)	0 (0.0%)	
		No	1,300 (99.9%)	6,505 (100.0%)	0.167
	Light therapy	Yes	65 (5.0%)	252 (3.9%)	
		No	1,236 (95.0%)	6,253 (96.1%)	0.065
	Antibiotic treatment	Yes	72 (5.5%)	249 (3.8%)	
		No	1,229 (94.5%)	6,256 (96.2%)	0.006
	Vitamin K	Yes	1,292 (99.3%)	6,454 (99.2%)	
		No	9 (0.7%)	51 (0.8%)	0.862
	BCG vaccination	Yes	212 (16.3%)	1,023 (15.7%)	
		No	1,089 (83.7%)	5,482 (84.3%)	0.618
	Hypothyroidism screening	Yes	1,278 (98.2%)	6,277 (96.5%)	
		No	23 (1.8%)	228 (3.5%)	0.001
	Metabolic disorder screening	Yes	55 (4.2%)	106 (1.6%)	
		No	1,246 (95.8%)	6,399 (98.4%)	< .001
Postterm	NICU	Yes	3 (6.7%)	14 (6.2%)	
		No	42 (93.3%)	211 (93.8%)	1
	Hospital transfer	Yes	2 (4.4%)	1 (0.4%)	
		No	43 (95.6%)	224 (99.6%)	0.073
	Respirator care	Yes	1 (2.2%)	0 (0.0%)	
		No	44 (97.8%)	225 (100.0%)	0.167
	Intubation	Yes	1 (2.2%)	1 (0.4%)	
		No	44 (97.8%)	224 (99.6%)	0.306
	Blood transfusion	Yes			
		No	45 (100.0%)	225 (100.0%)	1
	Light therapy	Yes	1 (2.2%)	9 (4.0%)	
		No	44 (97.8%)	216 (96.0%)	1
	Antibiotic treatment	Yes	4 (8.9%)	7 (3.1%)	
		No	41 (91.1%)	218 (96.9%)	0.091
	Vitamin K	Yes	45 (100.0%)	220 (97.8%)	
		No	0 (0.0%)	5 (2.2%)	0.594
	BCG vaccination	Yes	5 (11.1%)	42 (18.7%)	
		No	40 (88.9%)	183 (81.3%)	0.284
	Hypothyroidism screening	Yes	45 (100.0%)	222 (98.7%)	
		No	0 (0.0%)	3 (1.3%)	1
	Metabolic disorder screening	Yes	1 (2.2%)	7 (3.1%)	
		No	44 (97.8%)	218 (96.9%)	1

very preterm = gestational weeks 24–29; preterm = gestational weeks 30–34; near term = gestational weeks 35–37; term = gestational weeks 38–41; postterm = gestational weeks 42+

Abbreviations: ACT, antenatal corticosteroid therapy; BCG, bacille Calmette-Guérin; NICU, neonatal intensive care unit; PSM, propensity score matching.

**Table 5:** Comparison of hospitalization status beyond the first 7 days of life by ACT for the PSM subsamples stratified by timing of birth and infant sex.

Sex	Timing of Birth	Hospitalized Beyond 7 days	Treated *n* (%)	Control *n* (%)	Fisher exact *P*
Male	Very preterm	Yes	81 (100.0)	81 (100.0)	
		No	0 (0.0)	0 (0.0)	
	Preterm	Yes	452 (91.9)	790 (89.7)	
		No	40 (8.1)	91 (10.3)	0.213
	Near term	Yes	113 (32.0)	346 (19.8)	
		No	240 (68.0)	1,405 (80.2)	< .001
	Term	Yes	41 (6.1)	101 (3.0)	
		No	633 (93.9)	3,265 (97.0)	< .001
	Postterm	Yes	4 (18.2)	0 (0.0)	
		No	18 (81.8)	102 (100.0)	0.001
Female	Very preterm	Yes	60 (95.2)	62 (100.0)	
		No	3 (4.8)	0 (0.0)	0.244
	Preterm	Yes	373 (91.4)	551 (87.0)	
		No	35 (8.6)	82 (13.0)	0.034
	Near term	Yes	137 (41.3)	299 (18.0)	
		No	195 (58.7)	1,359 (82.0)	< .001
	Term	Yes	32 (5.1)	69 (2.2)	
		No	593 (94.9)	3,058 (97.8)	< .001
	Postterm	Yes	0 (0.0)	6 (4.9)	
		No	23 (100.0)	117 (95.1)	0.59

very preterm = gestational weeks 24–29; preterm = gestational weeks 30–34; near term = gestational weeks 35–37; term = gestational weeks 38–41; postterm = gestational weeks 42+

Abbreviations: ACT, antenatal corticosteroid therapy; PSM, propensity score matching.

Very preterm, near-term, and term-born infants exposed to ACT were consistently smaller at birth than controls regardless of mode of delivery (S2–S4 Tables). Infants born by planned cesarean would have received ACT in accordance with guidelines 7 days prior to delivery. The largest deficit (342 g) in BWT was seen for near-term infants in this group.

## Discussion

In this population cohort study of 278,508 live-born singleton births, exposure to ACT was associated with smaller size at birth in comparison to unexposed infants. Matched analyses on background and medical characteristics as well as timing of birth showed smaller birth size among infants prenatally exposed to ACT, though no difference in birth size was detected for the infants with shortest (24–29 weeks) or longest (≥42 weeks) gestation in the stringent PSM analyses. Consistent differences were observed in BWT in matched analyses among preterm, near-term, and term infants, with the greatest reduction in BWT seen in preterm infants. Similarly, BL and HC were most compromised in the preterm group, and clinically significant reductions were also observed for the near-term and term groups. Observed reductions in size in relation to ACT were generally symmetrical, i.e., no difference in PI.

To our knowledge, this is the largest study conducted to analyze the impact of ACT on birth size—4,887 individuals in total—and far exceeds the number used in the latest Cochrane review database of RCTs and BWT [28] using evidence downgraded to moderate (because of uncertain risk of bias and limitations of study design). Thus, these findings provide clinical evidence in humans that confirms previous suspicions that ACT would reduce growth [14–16].

Our primary outcome was birth size, so to accurately examine differences, we stratified all analyses by timing of birth and controlled for all available significant background variables. ACT is indicated to prepare lung maturation in preterm infants; thus, infants born at term represent the group who unnecessarily received treatment. This group provides an indication of whether growth deficits are due to ACT exposure or potentially to poor fetal health precipitating ACT.

Term infants who were exposed to ACT showed significant and symmetrical reductions in all birth size measurements (BWT, BL, and HC), indicating that even earlier treatment (at the time of threatened preterm birth) had a lasting impact on growth trajectory. The overall 89.38 g difference in BWT among the term group corresponds to a loss of about 5 days of optimum growth (at term) according to recent international standards [29]. In comparison, BWT reductions of 90–150 g are associated with maternal smoking [30].

The 5-minute Apgar scores indicated that infants born preterm, regardless of ACT treatment, were generally equally vigorous. At term, Apgar scores were slightly higher for unexposed male infants, whereas female infants had lower scores when born very preterm, near term, at term, or post term. These results are worthy of further investigation to determine whether ACT would have a differential impact on Apgar scores dependent on fetal sex. Recent evidence shows distinct biological pathways linking fetal sex with various complications associated with restricted growth [31].

It is noteworthy that both male and female infants exposed to ACT were more likely to receive medical interventions and to remain hospitalized beyond 7 days, though the overall number of hospitalized infants was small. Our matched and stratified analyses show that there were no differences in care regarding RDS—e.g., neonatal intensive care unit (NICU), respirator care, or intubation—for the very preterm or postterm infants whether or not they had received ACT. In fact, these medical interventions related to RDS were more likely among ACT-treated infants than controls born preterm, near term, or at term. These findings suggest that ACT was confounded by medical indication. Moreover, it is also possible that reduced growth associated with ACT contributes to compromised neonatal health, thus leading to more medical interventions. These results are similar to those recently reported [8] showing that as the period between ACT and birth increased (i.e., birth near term or at term), so too did the risk of neonatal respiratory morbidity and hospitalization. The intention of prophylactic use of ACT is to increase vitality at birth and reduce morbidity. In contrast, our results of matched comparisons using PSM subsamples and timing of birth indicate that the ACT group received more care. A large multinational cluster RCT of low- and middle-income countries reported higher, rather than lower, mortality among the preterm infants prenatally exposed to ACT [32]. Further, a meta-analysis examining ACT for elective cesarean section of term births concluded that although a small yet significant effect of ACT for prevention of neonatal morbidities was observed, the authors strongly cautioned against routine use of ACT because of other potential harms [33].

Reductions in size of infants also consistently differed by mode of delivery and ACT. For preterm, near-term, and term-born infants, we observed a graded difference in BWT reductions, with the highest growth deficits for the unplanned cesarean deliveries, followed by planned cesarean, and the least reductions in the vaginally delivered group. These findings merit further investigation to disentangle the effects.

HC in relation to ACT is seldom reported. The few studies that do report HC report similar deficits [14,34–35]; HC at birth reflects brain growth throughout gestation and has important implications for neurodevelopment [36]. Basset and colleagues [37] report ACT was protective for neurodevelopment only for infants with larger HC, indicating larger HC at birth confers protection for preterm infants. A large prospective follow-up found an association between ACT exposure and poorer scores on validated mental health screeners in childhood [38]. Smaller HC for preterm, near-term, and term infants, as shown by our data, may have long-term implications.

BL is strongly associated with adult height, more so than other birth measurements [39], and adult height in turn confers risk of death from several major chronic diseases [40]. We consistently found shorter BL among ACT-exposed infants, which bears clinical significance for health over the life course.

### Study strengths and limitations

This study has several unique strengths afforded by the use of objective population-based register data. As far as we know, no other study is based on such a large sample or has focused on birth size as the primary outcome. Though observational in nature, the size of this study provided the opportunity to carefully match infants on baseline characteristics that could have influenced clinical decisions to treat, including risk factors/complications (e.g., preeclampsia) that can lead to both spontaneous and elective preterm birth as well as intrauterine growth retardation. The PSM technique enabled us to limit possible bias and include five matched controls per patient for most analyses, which has been found to be the optimum number for bias reduction [27]. In this way, difference between groups on severity of gestational complications is minimized, as five controls are compared to one patient.

We stratified our analysis by gestational age. Stratification is the method of choice for controlling potential confounding and for identifying effect modification related to timing of birth [41], which would not be captured had we merely controlled for gestational age. Our stratified analysis revealed a dose-response-like association with greatest deficits in growth seen in all measurements (BWT, BL, and HC) for preterm infants and reducing in magnitude over time until eventually disappearing for infants born post term, thus suggesting a potential opportunity for catch-up growth in utero or changing contribution of medically indicated preterm births. At term, the growth deficits were still statistically and clinically significant, indicating that even though some degree of catch-up growth may have occurred, the impact of ACT was long-lasting. Regardless of analytic strategy (PSM or adjusted regression analyses), the results were consistent and in the same direction, thus indicating that collider stratification bias is not likely to operate [42]. Further, it is likely that because the sample size was so large, the effect of ACT on birth size was not attributable to bias in treatment allocation. In fact, the entire sample produced estimates of greater deficits in birth size, which are probably inflated as compared to the matched analyses. Thus, we provide here robust estimates.

An advantage of carrying out our study in Finland is the high-quality and uniform antenatal care that is offered free of charge to women residing in Finland. Most women have on average 15 antenatal care visits [43]. There are no sociodemographic differences in the uptake of care [43], so it is unlikely that complications were missed because of insufficient care by a more vulnerable group; on the contrary, poor pregnancy outcomes are associated with a higher number of visits, indicating greater surveillance [43]. Thus, confounding due to inequalities in access to care is highly unlikely in this sample. Our results show that there were no differences in socioeconomic status associated with treatment. This is particularly important because socioeconomic inequalities are likely to confound birth size yet are rarely mentioned in previous research. Understandably, the rate of preterm birth is low in Finland, as the standard of antenatal care is among the best in the world. This fact helps to discern well the impact of ACT from potential confounders related to access to care. However, countries where preterm rates are higher may also have care conditions that are less favorable for the preterm neonate who would benefit from ACT to promote survival. Indeed, care decisions should also consider issues of morbidity, neonatal care availability, and quality of life beyond survival.

We concentrated on live-born infants because our emphasis is on growth restriction among survivors. Stillbirths in high-income countries are also associated with avoidable maternal lifestyle factors such as obesity and smoking [44], factors that we controlled for in our analyses. Stillbirths can be related to major complications or malformations, which would make it very difficult to discern the impact of ACT on such infants.

Lack of detail in FMBR concerning ACT is the main limitation of this study. For example, we lacked information on the time lag between ACT and birth (which would have enabled examination of critical periods of growth), drug name, dose, or number of doses (which would enable drug-specific conclusions). Betamethasone remains biologically active for a period ranging between 36 and 54 hours after administration. Thus, the infants who were born shortly after ACT administration would be unlikely to receive the full effect of the drug. Any growth deficit would be due to compromised fetal health. In contrast, those born at least 3 days after treatment are likely to have had a full effect of the drug. Growth deficits in these infants would be due to both compromised health that led to treatment in the first place and the ACT itself. However, we expect that national guidelines were implemented that recommend a single dose of betamethasone and use tocolytic therapy to delay birth when needed. Data on serial ultrasound scans are not recorded in FMBR. Studies with serial ultrasound scanning would elucidate this issue by monitoring growth trajectory before and after ACT to determine the independent effects attributable to restricted growth due to poor health versus due to treatment. As seen in our DAG representation, unmeasured factors including genetic liability and lifestyle characteristics, such as health-related behaviors and mental health, are important factors to include. Thus, the information gained herein is useful for designing future cohort studies, which would not be restricted by population-based register data but would likely be of a smaller sample size.

Our data underline the need to establish clinical practices to predict parturition with greater accuracy to avoid inessential treatment, as nearly 45% of exposed infants were born at term. Our report should serve as an impetus for identifying techniques to more accurately predict preterm labor. Such advances have been preliminarily reported for a blood test that could cheaply and accurately predict parturition [45] and go beyond fibronectin tests, which are costlier and less often used worldwide. Appropriately targeting high-risk women for ACT would result in less medical over-intervention. Our report of consistent reductions in growth (BWT, BL, HC) coupled with increased medical interventions at birth for infants exposed to ACT calls for further study and reevaluation of clinical practice.

## Supporting information

S1 Table Comparison of birth size by ACT treatment versus no treatment using unadjusted and adjusted multiple regression analyses for the entire sample. ACT, antenatal corticosteroid therapy.
(DOCX)

S2 Table Comparison of birth size by ACT treatment for infants born by planned cesarean section, using PSM samples. ACT, antenatal corticosteroid therapy; PSM, propensity score matching.
(DOCX)

S3 Table Comparison of birth size by ACT treatment for infants born by unplanned/emergency cesarean section, using PSM samples. ACT, antenatal corticosteroid therapy; PSM, propensity score matching.
(DOCX)

S4 Table Comparison of birth size by ACT treatment for infants born by vaginal delivery, using PSM samples. ACT, antenatal corticosteroid therapy; PSM, propensity score matching.
(DOCX)

S1 Checklist STROBE checklist. STROBE, Strengthening the Reporting of Observational Studies in Epidemiology.
(DOCX)

## Abbreviations

ACT: antenatal corticosteroid therapy
BCG: bacille Calmette-Guérin
BL: birth length
BP: blood pressure
BWT: birth weight
DAG: directed acyclic graph
FMBR: Finnish Medical Birth Register
HC: head circumference
NICU: neonatal intensive care unit
PI: ponderal index
PSM: propensity score matching
RCT: randomized controlled trial
RDS: respiratory distress syndrome
STROBE: Strengthening the Reporting of Observational Studies in Epidemiology
