# Supplementary material for: Antenatal corticosteroid therapy (ACT) and size at birth: A population-based analysis using the Finnish Medical Birth Register
Source: PLoS Med. 2019 Feb 26;16(2):e1002746. doi: 10.1371/journal.pmed.1002746 (PMC6390995; doi:10.1371/journal.pmed.1002746)
Supplement: S1 Table — ACT, antenatal corticosteroid therapy. (DOCX) [file pmed.1002746.s001.docx]

S1 Table. Comparison of birth size by ACT treatment versus no treatment using unadjusted and adjusted^a^ mulitple regression analyses for the entire sample.

|  |  |  |  | Unadjusted Regression | | |  |  |  | Adjusted Regression | |  |
| --- | --- | --- | --- | --- | --- | --- | --- | --- | --- | --- | --- | --- |
| Timing of Birth | Measurements | Number of treated | Number of control | Point estimate | Std Err | P Value |  | Number of treated | Number of control | Point estimate | Std Err | P Value |
| very preterm | Birth weight (g) | 602 | 209 | -61.26 | 24.12 | 0.01 |  | 438 | 144 | -61.54 | 28.62 | 0.03 |
|  | Birth length (cm) | 538 | 185 | -0.70 | 0.30 | 0.02 |  | 386 | 132 | -0.68 | 0.35 | 0.05 |
|  | Ponderal index | 538 | 184 | -0.01 | 0.03 | 0.73 |  | 386 | 131 | -0.01 | 0.04 | 0.75 |
|  | Head circumference (cm) | 366 | 134 | -0.27 | 0.24 | 0.25 |  | 267 | 95 | -0.28 | 0.29 | 0.34 |
|  |  |  |  |  |  |  |  |  |  |  |  |  |
| preterm | Birth weight (g) | 1551 | 1953 | -232.90 | 17.24 | <.001 |  | 1140 | 1521 | -222.78 | 19.64 | <.001 |
|  | Birth length (cm) | 1415 | 1829 | -1.47 | 0.11 | <.001 |  | 1033 | 1432 | -1.42 | 0.13 | <.001 |
|  | Ponderal index | 1414 | 1829 | -0.03 | 0.02 | 0.05 |  | 1033 | 1432 | -0.04 | 0.02 | 0.02 |
|  | Head circumference (cm) | 1232 | 1639 | -0.89 | 0.08 | <.001 |  | 899 | 1287 | -0.83 | 0.08 | <.001 |
|  |  |  |  |  |  |  |  |  |  |  |  |  |
| near-term | Birth weight (g) | 908 | 18764 | -171.50 | 17.52 | <.001 |  | 685 | 14491 | -159.25 | 19.14 | <.001 |
|  | Birth length (cm) | 882 | 18507 | -0.79 | 0.08 | <.001 |  | 667 | 14285 | -0.75 | 0.09 | <.001 |
|  | Ponderal index | 882 | 18507 | -0.03 | 0.03 | 0.19 |  | 667 | 14285 | -0.02 | 0.03 | 0.38 |
|  | Head circumference (cm) | 849 | 18217 | -0.47 | 0.06 | <.001 |  | 647 | 14070 | -0.43 | 0.06 | <.001 |
|  |  |  |  |  |  |  |  |  |  |  |  |  |
| term | Birth weight (g) | 1756 | 238407 | -101.95 | 10.89 | <.001 |  | 1301 | 183825 | -91.62 | 11.86 | <.001 |
|  | Birth length (cm) | 1745 | 237403 | -0.44 | 0.05 | <.001 |  | 1291 | 183037 | -0.36 | 0.05 | <.001 |
|  | Ponderal index | 1745 | 237402 | -0.01 | 0.01 | 0.24 |  | 1291 | 183037 | -0.01 | 0.01 | 0.17 |
|  | Head circumference (cm) | 1730 | 236117 | -0.25 | 0.03 | <.001 |  | 1279 | 182046 | -0.21 | 0.04 | <.001 |
|  |  |  |  |  |  |  |  |  |  |  |  |  |
| post-term | Birth weight (g) | 69 | 14248 | -8.66 | 52.02 | 0.87 |  | 45 | 10591 | -15.87 | 60.96 | 0.79 |
|  | Birth length (cm) | 69 | 14175 | -0.25 | 0.22 | 0.25 |  | 45 | 10535 | -0.37 | 0.26 | 0.15 |
|  | Ponderal index | 69 | 14175 | 0.03 | 0.05 | 0.59 |  | 45 | 10535 | 0.04 | 0.07 | 0.61 |
|  | Head circumference (cm) | 69 | 14065 | -0.19 | 0.16 | 0.24 |  | 45 | 10451 | -0.14 | 0.18 | 0.44 |

^a^Adjusted for the same variables listed in Table 1

very preterm=gestational weeks 24-29

preterm=gestational weeks 30-34

near-term=gestational weeks 35-37

term=gestational weeks 38-41

post-term=gestational weeks 42+
