# Supplementary material for: Antenatal corticosteroid therapy (ACT) and size at birth: A population-based analysis using the Finnish Medical Birth Register
Source: PLoS Med. 2019 Feb 26;16(2):e1002746. doi: 10.1371/journal.pmed.1002746 (PMC6390995; doi:10.1371/journal.pmed.1002746)
Supplement: S2 Table — ACT, antenatal corticosteroid therapy; PSM, propensity score matching. (DOCX) [file pmed.1002746.s002.docx]

S2 Table.

Comparison of birth size by ACT treatment for infants born by Planned Caesarean Section using Propensity Score Matched (PSM) Samples

|  |  |  |  |  |  |  |
| --- | --- | --- | --- | --- | --- | --- |
| Timing of Birth | Measurements | Number of treated | Number of control | Point estimate | Std Err | P Value |
| very preterm | Birth weight (g) | 7 | 7 | -87.1 | 115.5 | 0.479 |
|  | Birth length (cm) | 7 | 7 | -1.43 | 1.6 | 0.4067 |
|  | Ponderal index | 7 | 7 | 0.05 | 0.11 | 0.6474 |
|  | Head circumference (cm) | 2 | 2 | -1.65 | 1.45 | 0.459 |
|  |  |  |  |  |  |  |
| preterm | Birth weight (g) | 93 | 114 | -255 | 109.2 | 0.0217 |
|  | Birth length (cm) | 80 | 97 | -1.55 | 0.57 | 0.008 |
|  | Ponderal index | 80 | 97 | 0 | 0.09 | 0.9835 |
|  | Head circumference (cm) | 61 | 68 | -0.43 | 0.42 | 0.3084 |
|  |  |  |  |  |  |  |
| near-term | Birth weight (g) | 150 | 750 | -342 | 61.54 | <.001 |
|  | Birth length (cm) | 142 | 696 | -0.83 | 0.27 | 0.0022 |
|  | Ponderal index | 142 | 696 | -0.16 | 0.03 | <.001 |
|  | Head circumference (cm) | 139 | 667 | -0.79 | 0.19 | <.001 |
|  |  |  |  |  |  |  |
| term | Birth weight (g) | 84 | 420 | -93.3 | 60.03 | 0.1239 |
|  | Birth length (cm) | 82 | 407 | -0.11 | 0.23 | 0.6182 |
|  | Ponderal index | 82 | 407 | -0.07 | 0.04 | 0.0688 |
|  | Head circumference (cm) | 80 | 391 | -0.41 | 0.19 | 0.0292 |
|  |  |  |  |  |  |  |
| post-term | Birth weight (g) | 1 | 5 | 1025 | . | . |
|  | Birth length (cm) | 1 | 5 | 2.2 | . | . |
|  | Ponderal index | 1 | 5 | 0.3 | . | . |
|  | Head circumference (cm) | 1 | 5 | 0.26 | . | . |

very preterm=gestational weeks 24-29

preterm=gestational weeks 30-34

near-term=gestational weeks 35-37

term=gestational weeks 38-41

post-term=gestational weeks 42+
