# Supplementary material for: Antenatal corticosteroid therapy (ACT) and size at birth: A population-based analysis using the Finnish Medical Birth Register
Source: PLoS Med. 2019 Feb 26;16(2):e1002746. doi: 10.1371/journal.pmed.1002746 (PMC6390995; doi:10.1371/journal.pmed.1002746)
Supplement: S3 Table — ACT, antenatal corticosteroid therapy; PSM, propensity score matching. (DOCX) [file pmed.1002746.s003.docx]

S3 Table. Comparison of birth size by ACT treatment for infants born by Unplanned/Emergency Caesarean Section using Propensity Score Matched (PSM) Samples.

| Timing of Birth | Measurements | Number of treated | Number of control | Point estimate | Std Err | P Value |
| --- | --- | --- | --- | --- | --- | --- |
| very preterm | Birth weight (g) | 77 | 77 | -63.2 | 43.52 | 0.1505 |
|  | Birth length (cm) | 61 | 61 | -0.66 | 0.58 | 0.2548 |
|  | Ponderal index | 61 | 61 | -0.03 | 0.05 | 0.5693 |
|  | Head circumference (cm) | 31 | 31 | -0.31 | 0.6 | 0.6143 |
|  |  |  |  |  |  |  |
| preterm | Birth weight (g) | 333 | 535 | -220 | 37.45 | <.001 |
|  | Birth length (cm) | 288 | 447 | -1.36 | 0.26 | <.001 |
|  | Ponderal index | 288 | 447 | -0.06 | 0.03 | 0.05 |
|  | Head circumference (cm) | 223 | 322 | -0.94 | 0.19 | <.001 |
|  |  |  |  |  |  |  |
| near-term | Birth weight (g) | 136 | 680 | -154 | 62.63 | 0.0153 |
|  | Birth length (cm) | 133 | 643 | -0.9 | 0.27 | 0.0014 |
|  | Ponderal index | 133 | 643 | 0.01 | 0.03 | 0.7771 |
|  | Head circumference (cm) | 124 | 575 | -0.79 | 0.18 | <.001 |
|  |  |  |  |  |  |  |
| term | Birth weight (g) | 125 | 625 | -142 | 56.16 | 0.013 |
|  | Birth length (cm) | 122 | 604 | -0.64 | 0.24 | 0.0083 |
|  | Ponderal index | 122 | 604 | -0.01 | 0.03 | 0.7752 |
|  | Head circumference (cm) | 120 | 585 | -0.44 | 0.15 | 0.0046 |
|  |  |  |  |  |  |  |
| post-term | Birth weight (g) | 8 | 40 | 15.1 | 173.4 | 0.933 |
|  | Birth length (cm) | 8 | 39 | -0.49 | 0.5 | 0.3594 |
|  | Ponderal index | 8 | 39 | 0.08 | 0.07 | 0.2732 |
|  | Head circumference (cm) | 8 | 37 | -0.15 | 0.59 | 0.804 |

very preterm=gestational weeks 24-29

preterm=gestational weeks 30-34

near-term=gestational weeks 35-37

term=gestational weeks 38-41

post-term=gestational weeks 42+
