# Supplementary material for: Antenatal corticosteroid therapy (ACT) and size at birth: A population-based analysis using the Finnish Medical Birth Register
Source: PLoS Med. 2019 Feb 26;16(2):e1002746. doi: 10.1371/journal.pmed.1002746 (PMC6390995; doi:10.1371/journal.pmed.1002746)
Supplement: S4 Table — ACT, antenatal corticosteroid therapy; PSM, propensity score matching. (DOCX) [file pmed.1002746.s004.docx]

S4 Table. Comparison of birth size by ACT treatment for infants born by Vaginal Delivery using Propensity Score Matched (PSM) Samples.

| Timing of Birth | Measurements | Number of treated | Number of control | Point estimate | Std Err | P Value |
| --- | --- | --- | --- | --- | --- | --- |
| very preterm | Birth weight (g) | 60 | 60 | 30 | 56.02 | 0.5943 |
|  | Birth length (cm) | 43 | 43 | 0.23 | 0.62 | 0.7125 |
|  | Ponderal index | 42 | 42 | -0.07 | 0.14 | 0.6318 |
|  | Head circumference (cm) | 24 | 24 | 0.69 | 0.7 | 0.334 |
|  |  |  |  |  |  |  |
| preterm | Birth weight (g) | 459 | 872 | -188 | 25.73 | <.001 |
|  | Birth length (cm) | 409 | 767 | -1.02 | 0.17 | <.001 |
|  | Ponderal index | 409 | 767 | -0.04 | 0.03 | 0.153 |
|  | Head circumference (cm) | 357 | 638 | -0.7 | 0.14 | <.001 |
|  |  |  |  |  |  |  |
| near-term | Birth weight (g) | 399 | 1995 | -111 | 26.37 | <.001 |
|  | Birth length (cm) | 392 | 1943 | -0.61 | 0.13 | <.001 |
|  | Ponderal index | 392 | 1943 | 0 | 0.02 | 0.8147 |
|  | Head circumference (cm) | 384 | 1887 | -0.33 | 0.08 | <.001 |
|  |  |  |  |  |  |  |
| term | Birth weight (g) | 1092 | 5459 | -79 | 15.01 | <.001 |
|  | Birth length (cm) | 1087 | 5414 | -0.3 | 0.07 | <.001 |
|  | Ponderal index | 1087 | 5414 | -0.01 | 0.01 | 0.1933 |
|  | Head circumference (cm) | 1079 | 5349 | -0.16 | 0.05 | 0.0005 |
|  |  |  |  |  |  |  |
| post-term | Birth weight (g) | 36 | 180 | -44.8 | 81.38 | 0.5856 |
|  | Birth length (cm) | 36 | 180 | -0.6 | 0.33 | 0.0818 |
|  | Ponderal index | 36 | 180 | 0.05 | 0.03 | 0.1008 |
|  | Head circumference (cm) | 36 | 178 | -0.36 | 0.26 | 0.1782 |

very preterm=gestational weeks 24-29

preterm=gestational weeks 30-34

near-term=gestational weeks 35-37

term=gestational weeks 38-41

post-term=gestational weeks 42+
